# Hybrid Imaging During Transcatheter Structural Heart Interventions

**DOI:** 10.1007/s12410-015-9349-6

**Published:** 2015-07-16

**Authors:** Patric Biaggi, Covadonga Fernandez-Golfín, Rebecca Hahn, Roberto Corti

**Affiliations:** Heart Clinic Zurich, Witellikerstrasse 40, 8032 Zurich, Switzerland; Ramón y Cajal University Hospital, Carretera de Colmenar Km 9.400, 28034 Madrid, Spain; Columbia University Medical Center, 177 Fort Washington Avenue, New York, NY 10032 USA

**Keywords:** Fusion imaging, EchoNavigator, DynaCT, Echocardiography, computed tomography, cardiac magnetic resonance imaging, Fluoroscopy, X-ray, TAVR, MitraClip, left atrial appendage, Mitral valve, aortic valve, paravalvular leak, Percutaneous, Structural heart disease, Heart team, Interventions, Multimodality image integration

## Abstract

Fusion of different imaging modalities has gained increasing popularity over the last decade. However, most fusions are done between static rather than dynamic images. In order to adequately visualize the complex three-dimensional structures of the beating heart, high-temporal and spatial image resolutions are mandatory. Currently, only the combination of transesophageal echocardiography with fluoroscopy allows real-time image fusion of high quality during structural heart disease (SHD) interventions. The use of markers as well as real-time image overlay greatly facilitates communication between SHD team members and potentially increases procedural success while reducing radiation dose and use of contrast. However, to date there is only limited evidence that fusion imaging improves safety and outcomes of SHD interventions. This review highlights the benefits of fusion imaging during SHD interventions such as transseptal puncture and closure of atrial septal defects and left atrial appendage as well as interventions on the mitral and aortic valve.

## Background

Clinically significant valvular heart disease increases with advancing age, reaching a prevalence of 11.7 % of those aged 75 years or older [1]. Surgery is indicated in many of these patients, but the perioperative mortality and morbidity risk increases in this aging and often comorbid population [2]. Numerous less invasive therapies such as percutaneous or transcatheter interventions have recently been introduced for treatment of structural heart disease (SHD). Transcatheter aortic valve replacement (TAVR) has proven to be equally or more effective than surgical aortic valve replacement for high-risk surgical patients [3, 4]. New devices effectively close the left atrial appendage and thus reduce the risk of thromboembolic complications in atrial fibrillation and even reduce mortality [5]. More than 18,000 patients with mitral regurgitation have been treated for moderate to severe mitral regurgitation by percutaneous mitral valve repair using the MitraClip device (Abbott Vascular, Santa Clara, CA) [6, 7]. And already new transcatheter options such as percutaneous mitral annuloplasty ring implantation or transcatheter mitral valve replacement appear on the horizon [8, 9].

Historically, interventional cardiologists work with fluoroscopy as the main tool for real-time guidance of catheter-based therapy. However, one of the key factors in the tremendous success of SHD interventions is the ongoing development and clinical implementation of advanced cardiac imaging [10]. Since interventions in structural heart disease are performed on the beating heart, visualization of the relevant structures with means other than direct visual inspection by the surgeon is crucial. Advances in cardiac imaging with three-dimensional (3D) transesophageal echocardiography (TEE) and multislice computed tomography (MS CT) have proven particularly helpful in demonstrating the complex valvular morphology and in performing necessary pre-interventional precise measurements for planning and tailoring of percutaneous therapies [11, 12]. Up to now, images during SHD interventions are displayed on several screens, thus requiring extensive effort of coordination and communication between imagers and interventionalists.

Fusion imaging projects echocardiographic images and guidance tools onto the fluoroscopy screen and may enhance workflow and improve procedural outcomes. This article will review the concept, literature, and current use of fusion imaging during various SHD interventions such as valvular repair or replacement as well as closure of paravalvular leaks or the left atrial appendage. Although not discussed, the use of this technology can also be applied to electrophysiology and congenital heart disease interventions.

## The Challenges During SHD Interventions

**Fig. 1:**
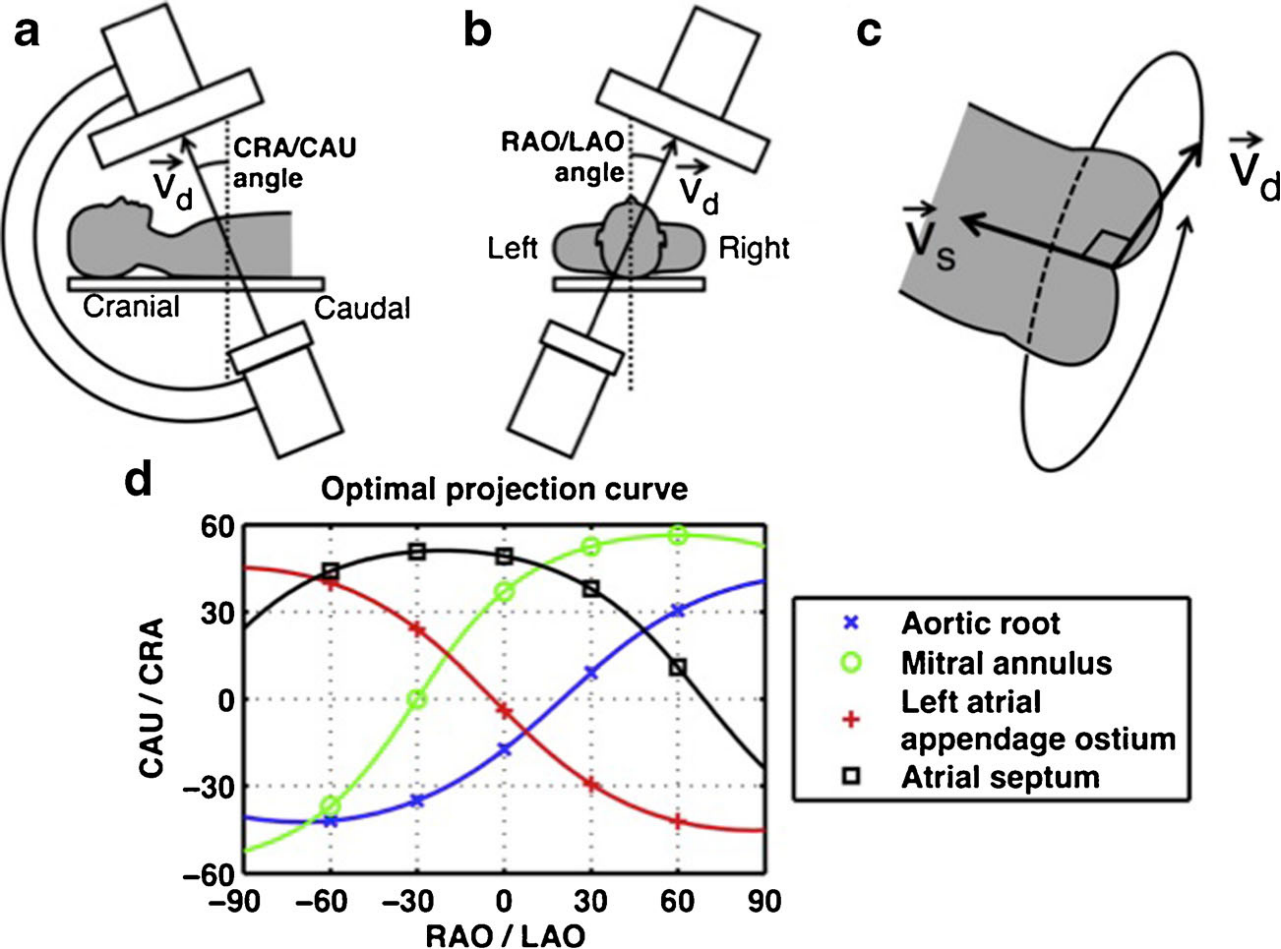
Geometry of the optimal projection curve during fluoroscopy. The vector joining the X-ray source and the center point of the detector is designated *v*_*d*_, and the vector pointing along a structure of interest is *v*_*s*_. **a**, **b** The angular system (cranial [*CRA*]/caudal [*CAU*] and right anterior oblique [*RAO*]/left anterior oblique [*LAO*] angles) used in fluoroscopy is described. **c** All vectors v_d_ perpendicular to vector v_s_ are optimal viewing angles. The optimal projection curve is the plot of the fluoroscopic angles of all vectors v_d_ for a particular structure of interest. **d** The optimal projection curve is shown for different cardiac structures. Reprinted from [17] with permission

SHD interventions are performed with specially designed catheters, guides, sheaths, and implantation tools. To perform successful interventions without causing any harm it is mandatory to use these tools with high precision. One of the challenges during structural heart interventions is to accurately visualize in real time the moving catheters and implant material within the beating heart. In addition, SHD interventions are complex and numerous guidelines recommend the implementation of a multidisciplinary SHD team rather than a single person [13–15]. The SHD team typically consists of cardiologists and cardiac interventionalists, a cardiac surgeon, cardiovascular imaging specialists, anesthesiologists, and specialized nurses. The action of the intervening specialists heavily depends on the images offered by the imaging specialist, who in turn needs to know the structures relevant to the interventionalist and what views are optimal for guiding the procedure. To complicate things further, the orientation of the projected images differs largely between imaging modalities. While the imaging windows of the TEE probe are typically limited to a narrow (although not fixed) view through the esophagus [16], the C-arm rotation in contrast allows multiple views of the same structure (Fig. 1) [17]. Thus, identifying structures simultaneously on echocardiographic and fluoroscopic imaging becomes complicated and prone to miscommunication. Furthermore, all imaging techniques have strengths and weaknesses, making the use of multiple imaging modalities necessary during interventions.

Hence, accurate identification of complex three-dimensional structures on multiple imaging modalities and effective communication of this anatomy within the SHD team become key factors for successful SHD interventions. To facilitate these tasks, real-time fusion imaging (i.e. fusion of the most commonly used imaging modalities into one) has recently been introduced [18, 19•, 20, 21].

## The Concept of Fusion Imaging

### Fusion of Static Images

Various fusion types of static imaging exist. Fusion of cardiac MS CT with single photon emission computed tomography has been used to correlate the coronary artery anatomy to the area of ischemia [22]. Lately, similarly fused images for the identification of ischemic areas have been achieved combining MS CT and echocardiography [23], different cardiac magnetic resonance modalities [24], and positron emission tomography (PET) with coronary angiography [25]. In the diagnostic workup of prosthetic heart valve infections, fusion of MS CT angiography with PET has proven helpful [26]. And for the selection of the correct size and type of prosthetic heart valve for TAVR, fusion of MS CT data with models of prosthetic implants has gained popularity [27]. These types of fusion, however, use two static images and are therefore not suitable during beating-heart, real-time SHD interventions.

### Fusion of Dynamic Images

**Fig. 2:**
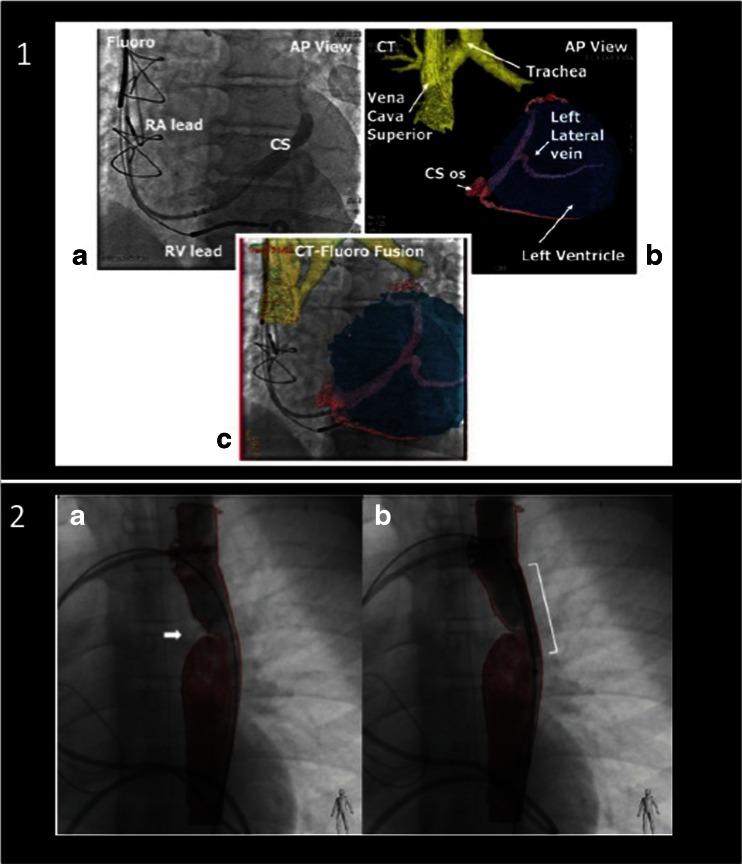
Fusion of computed tomography with fluoroscopy. Fusion of CT and fluoroscopy images has been used for interventions such as implantation of left ventricular leads for cardiac resynchronization (*1*, a–c) or for planning interventions in congenital heart disease such as coarctation of the aorta (*arrow* in *2*, *a* and *b*). *CS*, coronary sinus; *RV*, right ventricle; *RA*, right atrium. Reprinted from [19•, 30] with permission

For beating-heart interventions, systems fusing real-time images have been developed. Most systems use rapid CT performed in the hybrid intervention room and superimpose specific information (markers, overlay images) onto the fluoroscopy or angiography image (Fig. 2) [19•, 20, 21, 28–30]. The challenge during this type of hybrid fusion (fusion of a static with a dynamic image) is motion compensation for the beating heart and for breathing. This problem has been overcome by a software called EchoNavigator (Philips Medical Systems, Best, The Netherlands), which fuses real-time (“live”) images from TEE and fluoroscopy [18, 31, 32••].

### Real-Time Fusion of Echocardiography and Fluoroscopy

**Fig. 3:**
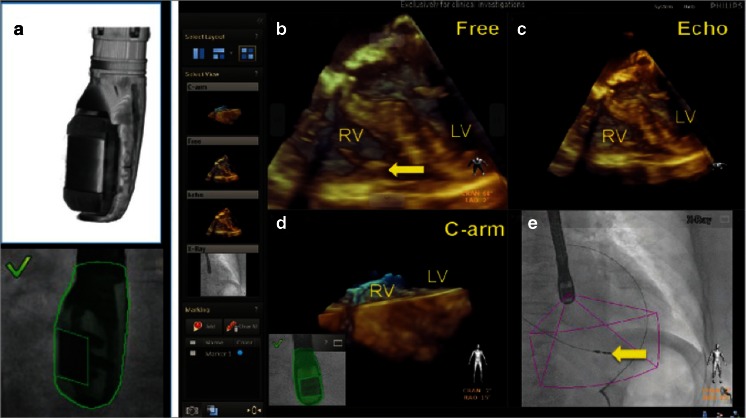
Real-time fusion of echocardiography and X-ray (fluoroscopy). Co-registration of X-ray and TEE is performed by fluoroscopic acquisition of the TEE probe in two angulated projections. The algorithm recognizes the position of the TEE probe by comparing the X-ray data with the acquired ultra-high-resolution volumes from the 3D model (**a**). The four images displayed simultaneously (**b**–**e**) by the EchoNavigator system are described as follows: **b** free rotated TEE image: this view can be freely manipulated by a mouse at the table site. **c** Echo image: this is the standard TEE projection as it appears on the echocardiographer’s screen. **d** The C-arm gantry view is the echocardiographic image orientated in the same plane as the X-ray view. **e** Finally, the fluoroscopy shows the angiographic view with the echocardiographic image volume displayed onto the X-ray view. The *yellow arrow* indicates the tip of a right ventricular pacemaker lead. *RV*, right ventricle; *LV*, left ventricle. Reprinted from [18] with permission

**Fig. 4:**
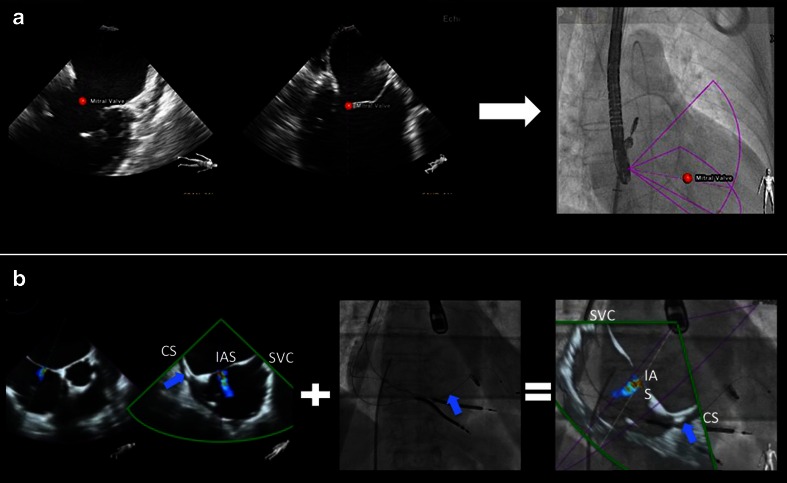
Tools in fusion imaging: markers (**a**) and overlay imaging (**b**). During the MitraClip intervention, the main mitral lesion can be labeled by the echocardiographer using a marker (*red dot* placed on the mitral valve). This marker is automatically demonstrated in real time on the fluoroscopy image (**a**). Using the overlay mode (**b**, showing an atrial septal defect), the echocardiographic image is overlaid on top of the fluoroscopy image in the correct position and angulation. The interventional cardiologist can choose between the purple and the green echo views given by xPlane echocardiography. In this case, the *green* view better demonstrates the anatomy for puncture purposes. The overlay mode highlights the orientation difficulties that SHD teams face during an intervention. In the modified TEE bicaval view, the superior vena cava is located to the right, while the coronary sinus is to the left, connected by the horizontally orientated interatrial septum. During fluoroscopy for transseptal puncture, the orientation of the C-arm is mostly neutral (in our example, LAO 13.5°, CRAN 0°). This leads to a completely different orientation: the superior vena cava is cranial, while the coronary sinus is at the bottom and to the lateral, with a more or less vertical interatrial septum. *SVC*, superior vena cava; *CS*, coronary sinus; *IAS*, interatrial septum. *Blue arrow*: depicting the left ventricular lead in the CS. **a** Adapted from Sündermann et al. [32••]

Real-time fusion of two or more cardiac imaging modalities of the beating heart is not a simple task. The key feature to enable correct real-time fusion is the co-registration of the echocardiography probe position with the intervention table and the angulation of the fluoroscopy C-arm [18, 33]. Special software is needed to recognize the TEE probe within the field of fluoroscopy view and to align its position with that of the C-arm. Once co-registration is successfully performed, the TEE probe and the fluoroscopy arm can be moved while image fusion is maintained (Fig. 3). Markers (dots or crosses) can be set to highlight important structures on the echocardiography image, and they are automatically displayed and updated in real time on the fluoroscopy image (Fig. 4a). Figure 4b demonstrates why the default image orientation of different imaging modalities can be confusing and how overlay imaging assists SHD teams in overcoming such challenges. The ability to overlay color Doppler images additionally facilitates the identification of specific targets, improving the rapid, accurate identification of structural lesions.

## Use of Real-Time Fusion Imaging in SHD Interventions

### Precise Transseptal Puncture

**Fig. 5:**
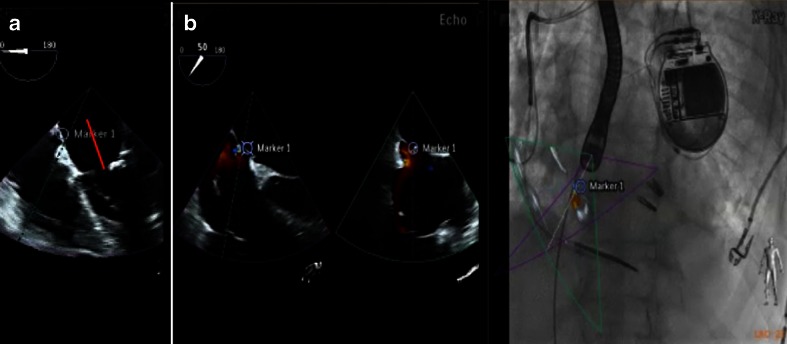
Correct puncture height (**a**) and safe passage (**b**) during transseptal puncture. During SHD interventions, the height of the transseptal puncture is often crucial. For the MitraClip procedure, the septum should be perforated at a distance of 4 cm from the mitral annular plane. This measurement is best achieved in a four-chamber view at roughly 0° (*marker* in **a**). However, an echo angulation of about 35°/125° in xPlane best shows the aortic root and the bicaval view, enabling safe and precise puncture. Using fusion imaging, the precise site chosen in the four-chamber view is also demonstrated in the aortic root short axis view (**b**, *marker 1* on echo and overlay image)

A targeted, precise, and safe puncture of the interatrial septum is the first important step for many SHD interventions. Depending on the procedure type, the puncture site should be inferoposterior (i.e., for percutaneous closure of the left atrial appendage [LAA]) or anterosuperior at a level of 4 cm above the mitral annulus (MitraClip implantation). In the latter case, the optimal puncture site is identified in the TEE four-chamber view (or roughly 0°) where the required distance can easily be measured (Fig. 5a). For the perforation however, the TEE angle is increased to roughly 45° and the simultaneous biplane or multiple plane function activated. The bicaval view is then used as an overlay on top of the fluoroscopy. This ensures a fast but nevertheless safe and very precise puncture of the interatrial septum (Fig. 5b).

### PFO/ASD Closure

**Fig. 6:**
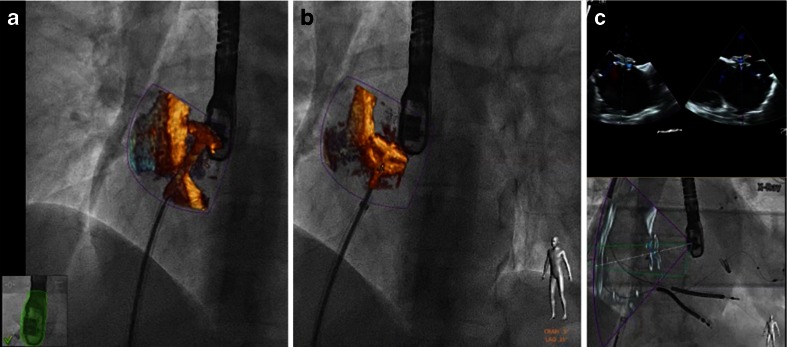
Fusion imaging guided ASD closure: 2D and 3D real-time TEE overlay on fluoroscopy. Using fusion imaging, the location of the sheet orifice is constantly visualized and the left atrial disc is released under direct vision (**a**). The right atrial disc is opened under echo-guidance (**b**). Color Doppler echocardiography overlay helps to determine correct development, localization, and function of the device without the use of contrast agent (**c**, different patient than **a** and **b**)

Many centers do not use echocardiographic guidance for patent foramen ovale (PFO) or atrial septal defect (ASD) closure [34, 35]. If the PFO channel is however long and rather narrow, wire passage can be time consuming. Using a marker on the fused fluoro-echo image facilitates this passage. In addition, a PFO and small ASDs can coexist. Without echocardiographic imaging, there is a high likelihood that the wire will pass the septum in a non-targeted manner. To achieve complete closure, however, anatomic knowledge is mandatory. Overlay image and/or the use of a marker ensure passage of the correct perforation and lead to complete ASD/PFO closure. Overlay imaging supports fast and safe deployment of the “left atrial umbrella” since there is constant control of the correct position of the sheet orifice within the left atrium (Fig. 6a, b). Using overlay imaging, this procedure can be performed without the use of contrast agents (Fig. 6c).

### Mitral Valve Interventions

#### Percutaneous Mitral Valve Repair Using the MitraClip

**Fig. 7:**
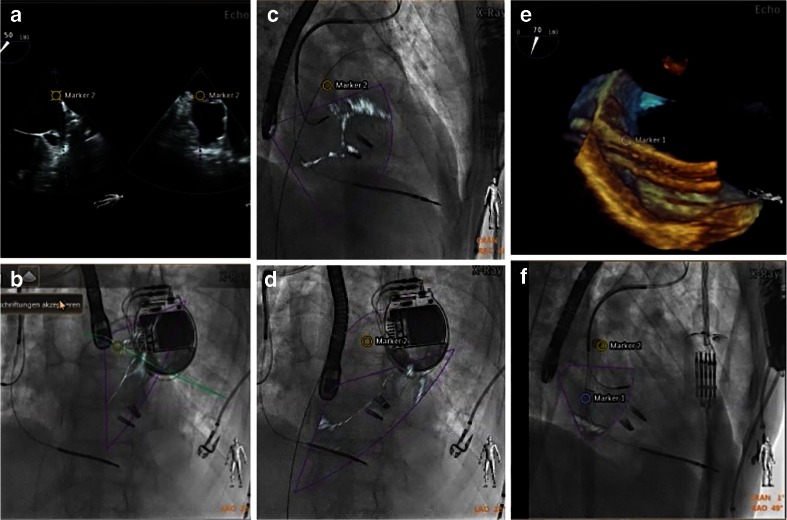
Fusion imaging may improve safety during SHD procedures. On the TEE image, a landmark (*marker 2*) is placed to identify the warfarin ridge on fluoroscopy (**a**, **b**). During the procedure, this critical landmark may not always be visible on echocardiography. Nevertheless, it is shown on the fluoroscopy image, with the position updated depending on the C-arm angulation (**c** LAO 44°, CRA 9°; **d** LAO 46°, CRA 12°). Such markers ensure safe and precise passage of a large catheter through the interatrial septum (**e**, *marker 1*) and may raise caution when approaching them (**f**, *marker 2*)

**Fig. 8:**
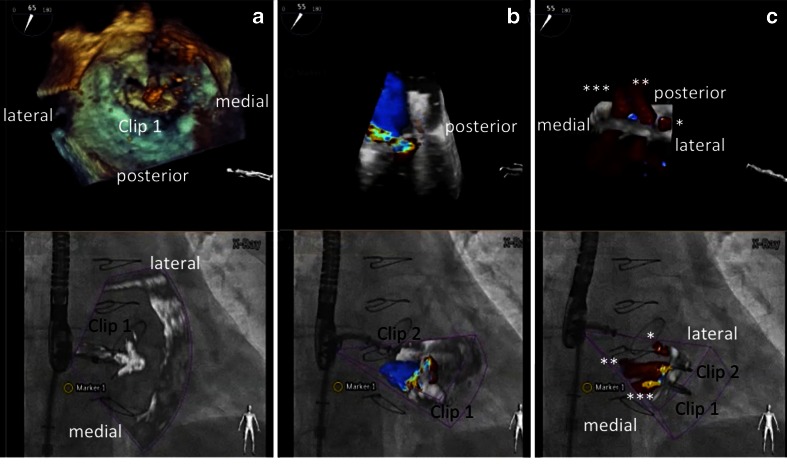
Overlay of 3D real-time echocardiography and fluoroscopy during a MitraClip procedure. **a**–**c** demonstrate step by step the insertion of clip 1 and 2 (each with 3D real-time echo on *top*, fusion image at the *bottom*). **a** Orientation of the clip relative to the mitral valve leaflet margin after clip opening. **b** Residual moderate mitral regurgitation after insertion of clip 1 in the medial part of the segments two. A second clip is approaching the valve. **c** Residual mild mitral regurgitation with three jets (denoted with *single*, *double*, and *triple asterisks*) after the insertion of the second clip

During MitraClip (MC) intervention, a steerable 24F sheet is used for the passage of the MC delivery system. Maneuvering such a device harbors dangers such as accidental puncture of the aortic root as well as perforation of the left atrial wall. Precision during the MC procedure is thus key for a safe and successful intervention, and the use of fusion imaging has turned this complicated procedure into a safe and effective one [31]. The first critical step is the targeted transseptal puncture as mentioned above. Once the interatrial septum is perforated and the sheet in place, steering the MC delivery system down to the mitral valve can be challenging on two-dimensional fluoroscopy. Erroneous steering may lead to long radiation and procedure time and potentially damage the left atrium free wall. Fusing live 2D and 3D echocardiography with fluoroscopy is safe and feasible in most patients and shows a trend towards reduction of fluoroscopy and procedure time [32••]. The use of the real-time overlay function for transseptal puncture (Fig. 5) and markers to identify the warfarin ridge (Fig. 7) as well as the target mitral lesion (Fig. 4a) is a key step. Real-time overlay imaging can also be used to guide clip insertion in multiple clip procedures (Fig. 8).

#### Mitral Paravalvular Leak Closure

**Fig. 9:**
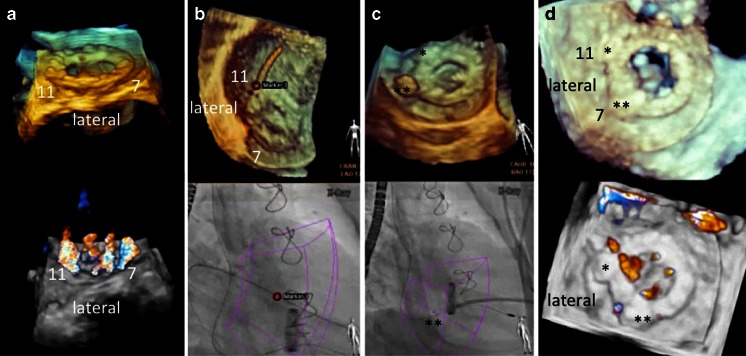
Fusion imaging during mitral paravalvular leak closure. 3D real-time echocardiography with and without color Doppler is helpful to determine the exact location and numbers of valvular and paravalvular jets (**a**). In this case, jets at 7 and 11 o’clock were considered the target lesions. **b** A *red marker* was used to locate the lesion at 11 o’clock (*upper image*), guiding the wire passage of the correct leak under fluoroscopy (*bottom image*). **c** The same approach was used to close the lesion at 7 o’clock with a vascular plug (*double asterisks*). **d** Echocardiographic images of the final result showing vascular plugs (*single* and *double asterisks*) at 11 and 7 o’clock, respectively, and a markedly reduced paravalvular and unchanged transvalvular regurgitation compared to **a**

Closure of paravalvular mitral regurgitation post mitral valve surgery can be performed with a transseptal or transapical approach. The advantage of fusion imaging for the transseptal approach is described above. For the transapical approach, identification of the optimal perforation site by echocardiography can help to achieve favorable “angles.” Fusion of fluoroscopy and echocardiography (Fig. 9) or fluoroscopy and DynaCT (Siemens AG, Forchheim, Germany) [21] has been used for the transapical approach. Real-time echo/fluoro fusion is used for the identification of the exact location of the paravalvular leak. This is particularly helpful in the presence of several leaks (Fig. 9a). Wire passage of the correct leak site is relatively easy once its location is marked on the fluoroscopy image (Fig. 9b). Closure of such leaks can be done without the use of contrast agents, a potentially relevant aspect in these often severely ill patients with renal insufficiency.

#### Transcatheter Mitral Valve Replacement

**Fig. 10:**
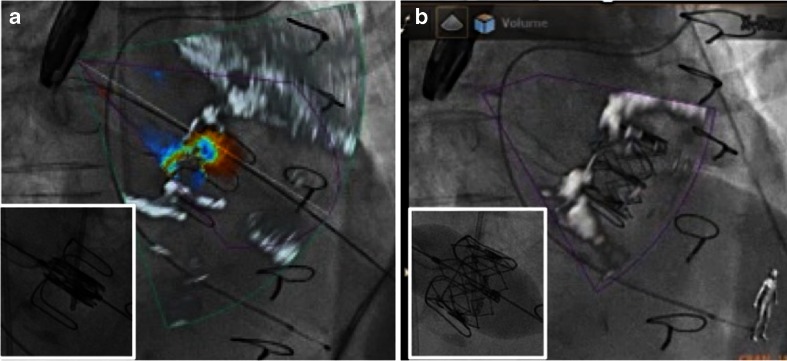
Fusion imaging before (**a**) and after (**b**) transapical mitral valve-in-valve replacement. **a** Fusion of 3D color Doppler transesophageal echocardiography with fluoroscopy: a failing 27-mm stented Edwards bioprosthesis in mitral position with flail of the lateral leaflet, leading to severe transvalvular mitral regurgitation. **b** After transapical valve-in-valve replacement, fusion imaging documents correct position of the implanted 26-mm Edwards Sapien XT bioprosthesis with complete leaflet coaptation and no residual regurgitation. *Small inlets* show fluoroscopy alone immediately before (**a**) and at the end (**b**) of the implantation of the Sapien XT prosthesis into the failing bioprosthesis

Current options for transcatheter mitral valve replacement include valve-in-ring (transseptal or transapical approach) and transapical valve-in-valve treatments [36–38]. The annuloplasty ring or valve prosthesis can be used as markers during intervention, and in contrast to the native valve and annulus, they are visible on fluoroscopy. Hence, fusion imaging is not as important for the placement and release of the prosthetic valve as it is for interventions in native soft tissue. Fusion imaging may however be used for choosing the proper access site (transseptal puncture and apical perforation as discussed above). Furthermore, the correct position and function of the implanted prosthetic valve is usually controlled by TEE, nicely demonstrated on fusion imaging (Fig. 10).

For the recently introduced mitral valve prostheses that will be used as percutaneous valve-in-native-valve procedure, fusion imaging will likely become equally important as with the MC procedure [37]. During transcatheter mitral valve replacement interventions, critical landmarks such as the mitral annulus or the aorto-mitral connection are not visible on fluoroscopy alone. In addition, the orientation of the prosthesis is of critical importance, and proper alignment will easily be achieved using the fused images. As this approach is expected to become almost equally relevant as TAVR, there is a great future for real-time fused imaging in this field.

### Aortic Valve Interventions

#### Transcatheter Aortic Valve Replacement

**Fig. 11:**
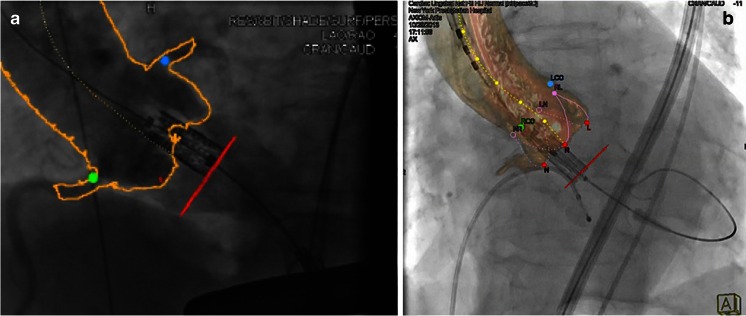
Fusion imaging during TAVR. Fusion imaging during transfemoral TAVR procedure. Both images show fusion of CT and fluoroscopy images. **a** The CT-gained boarders (marked *yellow line*) of the aortic root as well as the coronary ostia (*green* and *blue markers*) demonstrate perfect fusion with fluoroscopy. **b** A similar visualization is achieved using a semitransparent 3D model of the reconstructed aorta (based on CT data), overlaid on fluoroscopy. Both modalities enable deployment of the valvular prosthesis at the correct annular height (*red dots*). **a** Reprinted from [39] with permission

During TAVR, the valve prosthesis is implanted at the level of the (often invisible) aortic annulus. Fusion of DynaCT and fluoroscopy imaging provides a complete anatomic reference of the aortic root, including aortic annulus, sinuses of Valsalva, and coronary artery ostia [21, 39]. This can be demonstrated using markers (Fig. 11a) or complete overlay (Fig. 11b). Using CT and fluoroscopy overlay, implantation of the valve prosthesis in an anatomically correct orientation can be achieved [40••].

The shortcoming of current CT and fluoroscopy overlay is the inability of live fusion, i.e., there is insufficient motion compensation for the overlaid CT image. Once this problem can be solved, CT/fluoro overlay might become an alternative during TAVR in cases where the injection of contrast agent is contraindicated.

#### Aortic Paravalvular Leak Closure

**Fig. 12:**
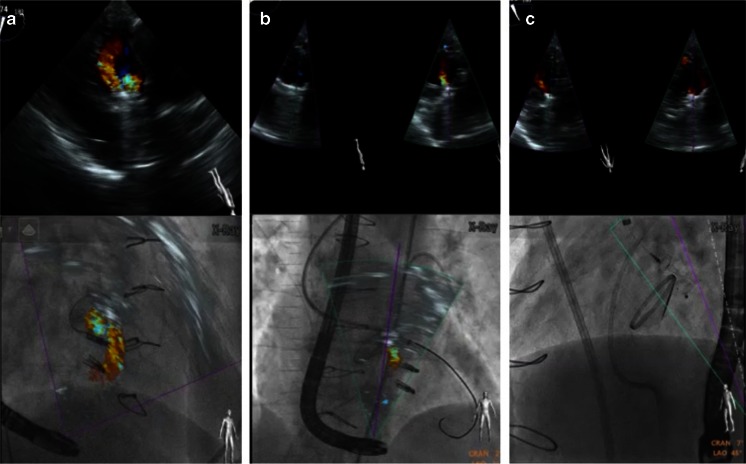
Fusion imaging during aortic paravalvular leak closure. **a** Due to extensive shadowing in midesophageal views, the amount of paravalvular leak can best be visualized in transgastric views (*top image*). Using proper C-arm angulation, co-registration of the TEE probe with the C-arm and hence fusion imaging even works during transgastric imaging (*bottom*). **b** Fusion imaging facilitates the wire and catheter passage through the leakage, immediately showing a reduction of aortic regurgitation as “proof” of correct passage. **c** Reduced paravalvular regurgitation after deployment of an Amplatzer Vascular Plug III ®

The challenge during closing of paravalvular leaks of aortic valve prostheses is the generally poor image quality by echocardiography. Extinction of crucial information due to shadowing by the prosthetic valve is common and especially prominent in patients with a mechanical prosthesis. While the metallic core and leaflets of the prosthetic valve can usually be seen on X-ray, the round structure makes the orientation difficult. Using markers and real-time overlay imaging facilitates localizing the perforation site and enables wire passage without the use of contrast. The reduction of aortic regurgitation after passing the paravalvular leakage with the catheter and after deployment of the vascular plug is demonstrated immediately (Fig. 12). The correct movement of the prosthetic leaflets can simultaneously be assured on fluoroscopy. Using proper C-arm angulation, the co-registration of the TEE probe with the C-arm also works during transgastric TEE.

### Left Atrial Appendage Occlusion

**Fig. 13:**
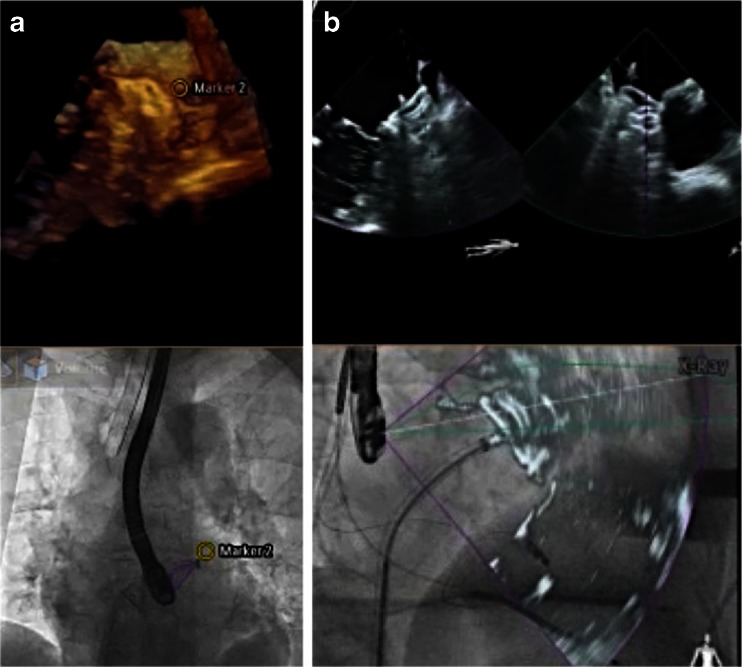
Fusion imaging during percutaneous left atrial appendage closure. **a** A marker in the echo image indicates the landing zone in the left atrial appendage, with the corresponding marker in the fluoroscopy image. **b** xPlane echocardiography (*top*) images demonstrate proper device placement in two planes, while simultaneous overlay imaging (*bottom*) ensures correct deployment and orientation of the (still attached) Amplatzer Cardiac Plug ® at the target zone

During percutaneous closure of the LAA, perforation of the LAA wall and laceration of the pulmonary artery can lead to pericardial tamponade and immediate death [41, 42]. The use of markers is helpful to localize the otherwise invisible LAA structures on fluoroscopy and prevent catastrophic complications. Such markers can be placed at the LAA orifice (at the level of the circumflex artery, Fig. 13a), the orifice of the left upper pulmonary vein (warfarin ridge), or the tip or bottom of the LAA. In addition, overlay imaging may help with the LAA orientation and ensure correct device position (Fig. 13b).

### Septal Alcohol Ablation in Hypertrophic Cardiomyopathy

**Fig. 14:**
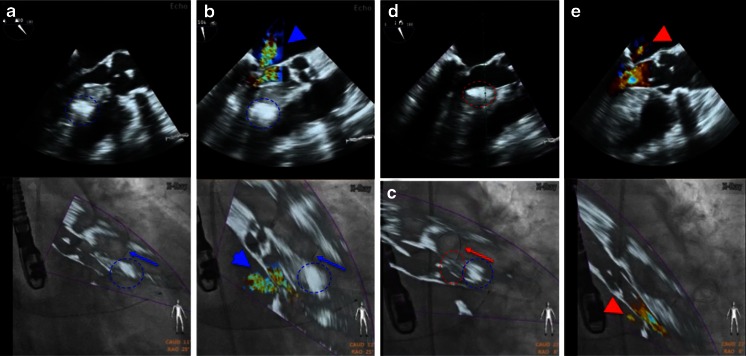
Fusion imaging during transcoronary ablation of septal hypertrophy (TASH). Fusion imaging during alcohol ablation of the second (**a**, **b**) and first (**c**–**e**) septal branch. **a** Wire (*blue arrow*) in the second branch after septal alcohol ablation, with the corresponding demarcated area (*blue dashed circle*). This however led to insufficient reduction of left ventricular outflow tract obstruction, as demonstrated by color Doppler imaging (mitral regurgitation due to systolic anterior motion, *blue arrow head* in **b**). **c** Placement of the wire into the first septal branch (*red arrow*), corresponding with a septal area (*red dashed circle*) just proximal to that of the second septal branch (*blue dashed circle*). **d** Demarcation of the proximal septum (*red dashed circle*) after alcohol infusion into the first branch. **e** Sufficient reduction of left ventricular outflow tract obstruction, demonstrated by markedly reduced mitral regurgitation an flow acceleration

Left ventricular outflow tract obstruction in hypertrophic cardiomyopathy can be removed either by surgical resection or by transcoronary alcohol ablation of septal hypertrophy (TASH) [43]. During TASH procedure, one of the most important steps is the identification of the septal branch perfusing the basal part of the hypertrophic septum. This can be straightforward in cases where one single branch is identified but more challenging when multiple small braches are present. In the case of several branches, the correlation between the branch and the septal muscle area can be nicely demonstrated by overlay imaging (Fig. 14).

## Limitation of Available Data on Fusion Imaging

This review on how to best use fusion imaging during different SHD interventions is largely based on the authors’ experience. This has two reasons: (1) until very recently, there was only one operation system commercially available for real-time fusion between dynamic images, and this system was installed only in very few hospitals. (2) As a consequence, there is very little data (in particular no randomized trial) proving the superiority of fusion imaging over the standard approach [32••]. Hence, whether the use of fusion imaging leads to a reduction of radiation dose, faster and safer interventions, and higher interventional success rates remains to be seen.

## Future Role of Fusion Imaging

Due to the constantly aging population in Western countries, aortic and mitral valve replacement therapies will be the leading interventions in SHD. As there is trend to expand percutaneous procedures to an intermediate or even low-risk population, procedural efficacy and safety will become even more important. This can only be achieved if all SHD team members dispose of an expert understanding of the three-dimensional structures of the heart and of fusion imaging techniques showing this anatomy in real time.

**Fig. 15:**
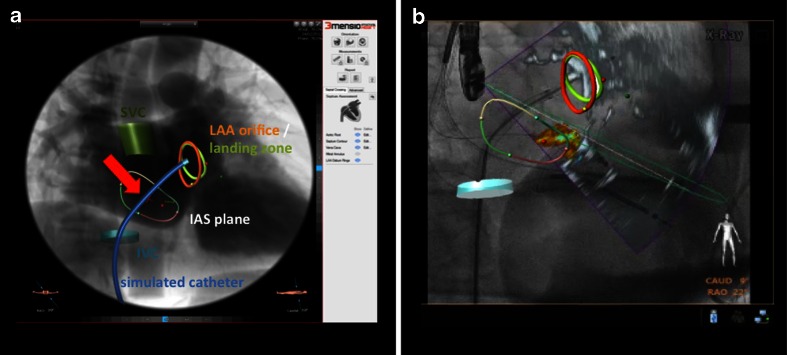
The future of fusion imaging. The future of fusion imaging for simulation and guidance of structural heart disease interventions. **a** Prototype software enables segmentation of relevant anatomical structures, such as the inferior vena cava or the interatrial septum. Ideally, a potential catheter could then be integrated into the image and determine the optimal perforation site in this patient (*red arrow highlighting red cross*). **b** Using motion compensation, this information should then be overlaid onto the real-time fusion of echo and fluoroscopy, potentially enabling a truly targeted and precise intervention. Please note: this figure was created by “fusing” images of two different existing software systems (“3mensio Structural Heart” and “EchoNavigator”), but does not represent currently available software. Reproduced with permission from 3mensio®

There is also room for improvement. Some anatomical structures are not well depicted by the current versions of real-time fusion. These include the irregularly shaped atria, the pulmonary valve and artery, as well as the left atrial appendage. In the near future, we expect increasing clinical importance of more advanced fusion imaging, such as overlay of static (but motion compensated), semitransparent three-dimensional low-dose CT images of the atria on fluoroscopy and echocardiography. Increasing computing capacities and more dedicated software will likely allow the use of real-time computer models simulating SHD interventions (i.e., simulation of the optimal [really targeted] septal puncture site, Fig. 15a) and overlaying such information during the actual intervention (Fig. 15b). Similar benefits could potentially be achieved for simulation of soft tissue reaction to deployment of prosthetic material, including deformation of mitral annulus during percutaneous mitral valve replacement, change in mitral valve geometry and residual mitral orifice area during MC interventions, or translocation of calcium and thus deformation of the aortic annulus during TAVR.

## Conclusion

Fusion of different imaging modalities has gained increasing popularity over the last decade. In order to adequately visualize complex three-dimensional cardiac structures, the beating heart asks for high-temporal and spatial image resolutions. Currently, only the combination of transesophageal echocardiography with fluoroscopy allows real-time image fusion of good quality during SHD interventions. The use of markers as well as real-time image overlay greatly facilitates communication between SHD team members and potentially increases procedural success while reducing radiation dose, procedure time, and contrast use. However, to date there is only limited evidence that fusion imaging improves safety and outcomes of SHD interventions.
